# Defining reasonable patient standard and preference for shared decision making among patients undergoing anaesthesia in Singapore

**DOI:** 10.1186/s12910-017-0172-2

**Published:** 2017-02-02

**Authors:** J.L.J. Yek, A.K.Y. Lee, J.A.D. Tan, G.Y. Lin, T. Thamotharampillai, H.R. Abdullah

**Affiliations:** 1 Department of Anaesthesiology, Singapore General Hospital, Singapore, 169608 Singapore; 2 Duke-NUS Medical School, Singapore, 169608 Singapore; 3 Yong Loo Lin School of Medicine, National University of Singapore, Singapore, 119228 Singapore; 4 Department of Dermatology, Singapore General Hospital, Singapore, 169608 Singapore; 5 Centre for Medical Ethics and Professionalism, Singapore Medical Association, Singapore, 169850 Singapore

**Keywords:** Informed consent, Anaesthesia, Material risk, Ethics, Communication, Shared decision-making

## Abstract

**Background:**

A cross-sectional study to ascertain what the Singapore population would regard as material risk in the anaesthesia consent-taking process and identify demographic factors that predict patient preferences in medical decision-making to tailor a more patient-centered informed consent.

**Methods:**

A survey was performed involving patients 21 years old and above who attended the pre-operative evaluation clinic over a 1-month period in Singapore General Hospital. Questionnaires were administered to assess patients’ perception of material risks, by trained interviewers. Patients’ demographics were obtained. Mann–Whitney *U* test and Kruskal-Wallis one-way analysis of variance was used. Statistical significance was taken at *p* < 0.05.

**Results:**

Four hundred fourteen patients were eligible of which 26 refused to participate and 24 were excluded due to language barrier. 364 patients were recruited. A higher level of education (*p* < 0.007), being employed (*p* < 0.046) and younger age group (*p* < 0.003) are factors identified in patients who wanted greater participation in medical decisions. Gender, marital status, type of surgery, and previous surgical history did not affect their level of participation. The complications most patients knew about were Nausea (64.8%), Drowsiness (62.4%) and Surgical Wound Pain (58.8%). Patients ranked Heart Attack (59.3%), Death (53.8%) and Stroke (52.7%) as the most significant risks that they wanted to be informed about in greater detail.

Most patients wanted to make a joint decision with the anaesthetist (52.2%), instead of letting the doctor decide (37.1%) or deciding for themselves (10.7%). Discussion with the anaesthetist (61.3%) is the preferred medium of communication compared to reading a pamphlet (23.4%) or watching a video (15.4%).

**Conclusion:**

Age and educational level can influence medical decision-making. Despite the digital age, most patients still prefer a clinic consult instead of audio-visual multimedia for pre-operative anaesthetic counselling. The local population appears to place greater importance on rare but serious complications compared to common complications. This illustrates the need to contextualize information provided during informed consent to strengthen the doctor-patient relationship.

**Electronic supplementary material:**

The online version of this article (doi:10.1186/s12910-017-0172-2) contains supplementary material, which is available to authorized users.

## Background

Informed consent is described as “voluntary authorization, by a patient or research subject, with full comprehension of the risks involved, for diagnostic or investigative procedures, and for medical and surgical treatment” [1]. It has traditionally been established upon the Bolam test, where duty of care was defined by acting in accordance ‘with a practice accepted as proper by a responsible body of medical men skilled in that particular art’ [1]. This was subsequently supplemented by the Bolitho qualification [2], where the court reserved the right to find a doctor negligent if he or she failed to meet a threshold test of logic and consistency.

There are many standards of risk disclosure that have since emerged from the days of the Bolam test, shifting away from the paradigm of a ‘reasonable physician standard’ to a ‘reasonable patient standard’, the ‘particular patient standard’ of material risk; and hybrids of the three. This was most recently evident in the English court decision, *Montgomery v Lanarkshire Health Board* [3], which stated that a doctor should take reasonable care to ensure that the patient is aware of any material risks involved in any recommended treatment, and of any reasonable alternative or variant treatments. ‘Material risk’ was referenced to whether a reasonable person, in the patient’s position, would be likely to attach significance to that risk, or whether the doctor is or should be aware that the particular patient would be likely to attach significance to it [2]. The Bolam-Bolitho approach still applies in the law of consent in Singapore. Bounded by *Dr Khoo James v Gunapathy d/o Muniandy* [4]*,* recent cases such as *Hii Chii Kok v Ooi Peng Jin London Lucien and another* [5]*, Chua Thong Jiang Andrew v Yue Wai Mun and another* [6] are examples where Bolam/Bolitho still applied. Notably, the Singapore High Court acknowledged *Montgomery* in both cases but in both instances was ruled that even under the *Montgomery* approach, the action would have been dismissed However, Singapore could possibly be reconsidering its position on the continued relevance of Bolam if the appropriate scenario arises.

The challenge to our practice is the limited information available regarding what the average patient undergoing general anaesthesia would constitute as a material risk; of which changes with time and varies across countries [7]. Lord Bridge in *Sidaway v Bethlem Royal Hospital* [8] of the English court stated that there is ‘no need to warn of the risks inherent in all surgery under general anaesthesia’, of which that are ‘relatively remote’. In contrary, Lord Diplock’s response was that as a ‘highly educated man of experience’, he should be warned of ‘all risks’.

Hence, we hope to understand our patients as studies have shown that better-informed patients report more realistic expectations, higher satisfaction, and demonstrate more treatment cooperation [3]. Changing legal and public expectations demand that we adapt our current practice and improve the information we provide to patients. Furthermore, patient satisfaction is not just about risk disclosure but more importantly, about shared decision-making [7]. Medical practitioners will have to adopt a more patient-centered approach by providing patients with information about material risks and empowering them to make their own decisions. Knowing what the average patient in Singapore would deem a material risk would be valuable to physicians in the proper education of patients.

Hence, this study aims to fill this knowledge vacuum by:

1. Understanding what the typical patient in the Singapore context would regard as material risks
2. Identifying predictive factors such as education level, occupation, demographics etc. that may influence a patient’s expectations for informed consent.
3. Understand the local population’s approach to risk disclosure, in terms of the nature and medium, and preferences to the medical decision-making process

## Methods

This cross-sectional study was conducted at Singapore General Hospital (SGH) in Singapore in April 2016. (CIRB Ref: 2015/3154). Patients who attended the pre-operative evaluation clinic for elective surgical procedures in April 2016 served as the study population. Study participants were selected by convenience sampling and were given a Patient Information Sheet prior to pre-anaesthetic consultation*.*

The selection criteria for patients included: having the capacity to participate in the study and comprehend the questionnaire as conducted by a trained interviewer; consenting to participate in the study; and being at least 21 years old and above. Patients with severe sensory disability that hampers understanding of questionnaire; severe cognitive impairment; or the inability to speak or comprehend English, Mandarin or Malay fluently were excluded from the study.

**Fig. 1:**
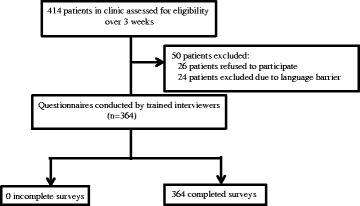
Methology of study

In order to estimate a population proportion with a 95% confidence interval assuming a population size of 15,000 and a conservative estimate of the sample proportion at 50%, the required sample size was 266 patients. The study managed to recruit 364 patients, which allowed for a 95% confidence interval and an alpha error of +/− 5% (Fig. 1).

**Fig. 2:**
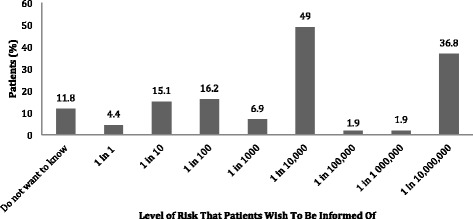
Collation of responses to extent of information desired in risks of daily living (Qn 1)

The trained interviewer administered the questionnaire upon verification of inclusion criteria and obtaining consent for participation in the study. The first eight items addressed demographics (Fig. 2), the next three items assessed risk perception, two items on risk communication format and the last item on preference for medical decision-making. Interviews were conducted in English and Mandarin, as well as Malay (with the aid of a translator). The questionnaire can be found under Additional file 1.

Statistical analysis was performed using IBM SPSS Statistics for Windows (V 23.0, IBM Digital Analytics, Armonk, N.Y., USA). Data was presented as mean ± SD after performing descriptive statistics and expressed in percentages. For variables with only two independent groups, Mann–Whitney *U* test was used; while Kruskal-Wallis one-way analysis of variance was used if there were more than two groups. Statistical significance was taken at *p* < 0.05.

## Results

### Demographics

This study included a total of 364 patients with demographics as summarized in Fig. 1. This was in concordance with the demographics of the general population who had undergone elective surgery at SGH in April 2016.

### Extent of information desired in risks of daily living

Question 1 of the interview studied patients’ preferences on the extent of information desired when risks of occurrence were tied to events of daily living, as summarized in Fig. 2. There was no correlation between patient demographics and the extent of information desired.

### Anaesthetic complications and risk perception

**Fig. 3:**
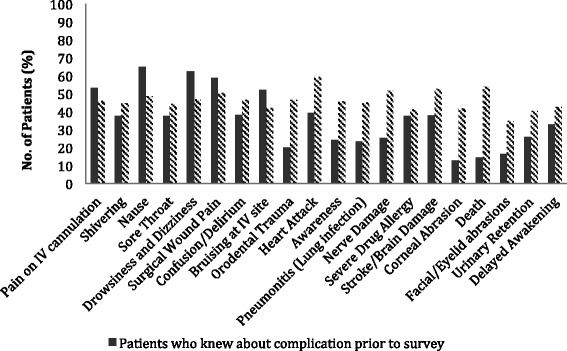
Collation of responses to participants’ responses towards risks of anaesthesia (Qn 2)

Figure 3 compiles the patients’ understanding of notable risks and complications of anaesthesia prior to the consult with the anaesthetist, and their desire to know any of them in greater detail (Question 3). Awareness of complications of general anaesthesia ranged from 12.9% (Corneal abrasions) to 64.8% (Nausea). The complications that patients were most familiar with were Nausea (64.8%), Drowsiness (62.4%) and Surgical Wound Pain (58.8%). In contrast, the least known risks were corneal abrasions (12.9%), death (14.5%), facial/eyelid abrasions (16.5%) and orodental trauma (20.1%). Heart Attack (59.3%), Death (53.8%) and Stroke (52.7%) ranked highest amongst complications that patients deemed were significant and wanted to be informed of. For every named complication, at least one- to two-third of patients considered it significant. Question 3 was an open-ended question which surveyed patients of any complications they had heard of that were not mentioned in the list stated in Question 2. There were no responses to the question.

### Extent of information desired in the anaesthetic consent

Question 4 of the survey studied patients’ preferences on the extent of information desired in the anaesthetic consent, as summarised in Fig. 2. Most patients (40.7%) wanted to know about all possible risks, 20.1% wanted to know only the dangerous risks, 19.8% wanted to know only the common risks, 2.7% wanted to know both the common and dangerous risks whereas 16.8% did not want to know any risks. Univariate analysis demonstrated that Age Group (*p* = 0.02), Level of Education (*p* = 0.007), and Employment Status (p = 0.04) had a significant association with the extent of information to be informed of the risks of general anaesthesia.

Using Spearman’s rank-order correlation, there was a strong, negative correlation with Age Group which was statistically significant (rs(8) = − 0.198, *p* = 0.0001); and a strong, positive correlation for the Level of Education (rs(8) = 0.174, p = 0.001). Binary regression analysis was employed to predict the probability of patients who would want to know their risks using the predictor variables of Age Group, Level of Education and Employment Status. The results were statistically significant (χ 2 = 49.642, p = 0.0001). Having a University education had a significant effect (*p* = .008). When corrected for confounding factors, a patient with a University education was 8.3 times more likely to want to know his or her risks compared to someone who does not have that level of education.

### Preference in quantifying risk

We next studied how patients preferred risks to be quantified during disclosure- in terms of description of risks, percentages or both, as summarized in Fig. 2. Thirty five percent of patients wanted both description and percentages of risks, 33% wanted description of risks only, whereas 18% wanted percentages only. More patients were comfortable with descriptions (68%) than percentages (53%). Fourteen percent of patients did not want any form of risk disclosure. Binary regression analysis was employed and did not show any statistical significance between demographics and preferred mode of quantifying risk.

### Medium of communication

**Fig. 4:**
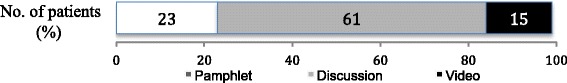
Collation of responses for patients' preferred medium of communication (Question 5B)

We then studied patients’ preferred medium of communication and additional decision aids if preferred – this was in the form of the options: ‘Discussion with the anaesthetist’, ‘Reading a pamphlet’, or ‘Watching a multimedia video’. Most patients preferred ‘discussion with the anaesthetist’ (61.3%) as compared to ‘reading a pamphlet’ (23.4%) or ‘watching a multimedia video’ (15.4%) as summarized in Fig. 4. Binary regression analysis was employed to predict the probability of patients’ preferred medium of communication. The results were statistically significant (χ 2 = 49.642, *p* = 0.000) for the option of ‘Discussion with the anaesthetist’. When holding all other variables constant, females were 1.8 times more likely than men to prefer discussion with their anaesthetist as their preferred medium of communication (*p* = 0.014).

### Approach to decision-making in healthcare

**Fig. 5:**
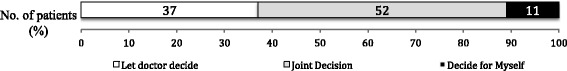
Collation of responses for patients' preferences in decision-making in healthcare (Question 6)

Question 6 of the survey depicts the level of responsibility for decision-making in healthcare that patients want. The responses collated for Question 6 have been re-categorized into “Doctor-dependent”, “Patient-dependent” level of decision-making, and “Making a Joint Decision” groups. The results are shown in Fig. 5: Most patients prefer to make a joint decision with their physicians (52.2%) as compared to allotting more responsibility to the physician (37.1%) or patients having full autonomy (10.7%).

Spearmann rho’s correlational analysis was used to test the hypothesis that patients who were less keen to know about any risks of anaesthesia (Question 4), are more likely to defer medical decisions to their doctors. We found a strong, positive correlation that is statistically significant (r_s_(8) = .0247, *p* = 0.0001) which supported the hypothesis.

Figure 5 reflects the correlation between patient demographics and the preferences of decision-making in healthcare. Patients were presented with options ranging from full patient autonomy, shared decision-making, to paternalistic decision-making process. Univariate analysis demonstrated that Paying Class (*p* = 0.002), Ethnicity (*p* = .01), Level of Education (*p* = 0.0002) and Age Group (*p* = 0.002) have significant correlations with patients’ medical decision-making. Correlational analysis performed between Level of Education and medical decision-making yielded a strong positive correlation (r_s_(8) = 0.285, *p* = 3.0 × 10^−8^). For Age Group, there exhibits a strong negative correlation (r_s_(8) = − 0.230, p = .00001). Ordinal regression analysis was employed to predict the probability of patients’ preference in medical decision-making. The ordered responses were the categories of dependant variable, and this was analysed against independent variables of age, education level, gender, ethnicity, marital status, household income and paying class (Private/subsidized). The results were statistically significant (χ 2 = 58.79, *p* = 0.002). Having no education (*p* = 0.009), holding only a primary education (*p* = 0.033) and Chinese ethnicity (*p* = 0.040) were associated with a ‘doctor-dependent’ decision.

## Discussion

### Risk perception

The survey first assessed patients’ risk perceptions in terms of life events (Question 1) and subsequently by anaesthetic complications (Question 2). A notable finding was that by specifying the nature of anaesthetic complication, patients’ risk perception heightened in contrast to when relating the risk to a life event, despite the fact that both the probabilities of occurrence were the same. The disparity between patients’ tolerance for quantitative risk information versus when tied to a qualitative risk information may suggest:

1. Risks are of significance to a patient not only by its frequency, but also by its severity. The definition of a material risk should be regardless of the infrequency of occurrence, but related to each individual’s circumstances [9–11].
2. Patients, regardless of age or level of education, may have difficulty understanding quantitative risk information in terms of probability, percentages and frequencies [9]. This is also supported by a recent study which found that as many as 40% of high school graduates could not perform basic numerical operations, such as converting 1% of 1000 – 10 of 1000. Termed “collective statistical illiteracy” [12], this is a major barrier to the interpretation of health statistics.
3. Risk information, if presented in probabilities of occurrence, could be perceived to be less threatening [12].
4. People may be dismissive of the risks taken daily due to denial, optimism, a feeling of immunity or invincibility. However, patients may feel more vulnerable under general anaesthesia as they are unconscious and thus have totally lost control of their circumstances [13].

### Prior knowledge and desire for information

Notably, more patients wanted to know about the severe, but rarer, complications such as heart attack, stroke and death compared with more common complications such as nausea/vomiting and shivering.

Moreover, most patients had little awareness of complications such as corneal abrasions, facial/eyelid abrasions and orodental trauma although these are the most common causes for litigation related to anaesthesia. This suggests the need to emphasize such complications during the consent process.

Contrary to physicians’ fear of distressing patients with information disclosure, our study has demonstrated that one in two patients would prefer to know these risks beforehand. This is congruent with other published studies that suggest that patients were able to ‘cope better with stressful medical procedures if warned about the distressing aspects beforehead’ [14]. Hence, it is not acceptable to assume that patients do not wish to know serious complications. The better way would be to establish patient’s role in decision-making and elicit their preferences.

Our findings suggest that the age group and education are factors that predict a greater appetite for knowledge. This is consistent with other studies and can be postulated that the younger and more educated patients have higher health literacy and hence can comprehend and retain information better [9, 10]. Therefore, this highlights the importance of improving health literacy, especially in the older and less educated patients, to achieve greater patient satisfaction.

Our results also reveal that there are patients, especially the elderly and uneducated population, who may wish to know little or nothing about their risks. Inevitably, there would be patients who cope better with reduced amounts of information where therapeutic privilege would be relevant, especially in the geriatric population. However, one must presume that all patients wish to be well informed about benefits and risks, and paternalistic assumptions are not acceptable. If the converse is true, then one should abide by the patient’s wishes and document this to be the case [15].

Additionally, for patients who have had previous surgeries, there was no apparent association between prior knowledge of anaesthesia and desire for information This could be attributed to multiple reasons such as failure to ensure completeness of information discussed, poor assessment of patient’s understanding or poor retention of medical information. Regardless, it highlights a gap in the consent-taking process where future studies can investigate methods to enhance patients’ understanding and ability to retain information.

### Mode of risk disclosure

The preferred mode of informed consent is still a discussion with the anaesthetist, in spite of the prevalence of printed and multimedia content [9]. This can be due to the trust and rapport built during the consultation process that would not have been possible through the interaction with the multimedia. Studies have also suggested that patient satisfaction may be linked to the engagement in discussion and decision-making process rather than full understanding of all information being provided [15]. This can be explained by the need for discussion through shared decision-making for trust to be built upon. This emphasizes how consent-taking should be an active process, in which the patient is not just a passive recipient of information, but engages actively in discussion and a process that builds rapport.

### Approach to decision-making in healthcare

As we progress towards a patient-centered care model, recognising the possible interplay of culture, education and patient demographics can assist physicians in individualized healthcare provision. Our results revealed that age, ethnicity and level of education were significant predictors of patients’ preferences in medical decision-making. In the local context, we can see that medical paternalism is still prevalent in the geriatric and Chinese population while the ‘autonomy paradigm’ is prevalent in the younger and more educated generation. Similar decision-making patterns were not prevalent in the other races, which may be due to the smaller sample size.

This could be attributed to the family and community structures in the Chinese family, which often emphasizes on family and physician authority; [16] or to a more developed culture of patient autonomy and associated legal directives in the younger generation, or both [17].

### Strengths and limitations of the study

First, the study was a qualitative than a quantitative analysis of the various complex factors in conducting an informed consent. Patient anxiety, trust and retention of information are as important as patients’ risk perspective. In addition, differences in expectations during informed consent between patients undergoing major and minor surgery were not elicited. The degree of actual understanding of consent was not assessed—while patients may choose discussion with the anaesthetist as their preferred mode of risk disclosure, it may not be the most effective method in achieving comprehension of information. Furthermore, our study does not evaluate the emotional impact of overwhelming patients with too much information in a short period of time. We also acknowledge that our study was not designed to evaluate the incremental effectiveness of decision aids such as multimedia and pamphlets that can offload time spent educating patients in the clinic and allow more dedicated time for discussion and interaction in the decision-making process.

Moreover, the study did not follow patients up postoperatively. Obtaining patients’ opinions of material risks postoperatively which incorporates their experiences of the surgical procedure and postoperative course may be more representative of true material risks, although this may be subject to recall bias. In addition, patient selection was selected using convenience sampling and hence results may not be extrapolated to the Singapore population.

### Implications for practice of anaesthesia

While it is commonplace to discuss complications with an incidence greater than 1% [16], as traditionally considered to define a material risk, we now understand that the severity of complications is of equal importance to patients as its frequency. This may not be common medical practice and clinicians should be made aware of patients’ perception of material risk.

Moving towards information technology may seem appealing due to benefits such as reduced consultation time and better understanding of risks;_17_ however, it may not improve patient satisfaction. Our results show how patients still prefer a discussion with the physician as the main mode of consent taking. This is consistent with the Institute of Medicine report that ‘people desire a patient experience that includes deep engagement in shared decision-making’ [18]. While the benefits of multimedia cannot be underplayed, multimedia cannot simply replace the consent-taking process, which allows for development of trust in the doctor-patient relationship.

Clinicians should be aware of the factors that may affect patients’ perspectives and responses to medical care as it would promote effective models of care. Notably, there are a small but significant proportion of patients who do not wish to be informed of any risks and prefer a paternalistic model of care. However, medical decision-making should not be prejudiced or stereotyped based on age or level of education. Socio-cultural context can shape the doctor-patient relationship but it does not speak of the complex issue of whether a patient’s social context is ethically preferable to the other [18].

Most importantly, we see a trend in the younger and educated patients who wish to make their own decisions as the decision is not solely based on medical considerations but that of ‘circumstances, objectives and values which he may reasonably not make known to the doctor but may lead him to a different decision from that suggested by a purely medical decision’ [3]. Even in Singapore, we can see that patients are now ‘widely regarded as persons holding rights, rather than passive recipients of the care of the medical profession’ and ‘consumers exercising choices’ [3]. The Singapore Medical Council’s Ethical Code and Ethical Guidelines 2016 [19] requires doctors to maintain patient’s ‘right to information and self-determination’ by keeping a patient adequately informed of his treatment options so that the patient can ‘make informed choices’. As clinical ethicists have proposed, the informed consent process should be centered about the patient’s expectations, cultures and values [14]. It is up to the clinician to invite the patient in the decision-making process and explore their preferences.

On the contrary, the applicability of *Montgomery* may give rise to defensive medicine and wastage of medical resources, as more time would be spent disclosing risks, regardless of its significance. As Singapore’s Chief Justice Menon [20] discussed at the opening address of the Legal Year 2016, it is important to ‘avoid a situation where practice of medicine comes to be adversely affected by the medical practitioner’s consciousness of the risk of malpractice liability’.

## Conclusion

Clinicians should recognise the increasing trend in patients’ preference for shared decision-making which should not only be based upon material risks, but also a decision-making process through which patients’ dignity is preserved and respect for their autonomy is maintained. Informed consent is a process that builds trust in a doctor-patient relationship. Enhancing informed decision-making can increase patient satisfaction and improve quality of care, both of which are cornerstones in the practice of good medicine.

## Additional file

Additional file 1: The file titled ‘Questionnaire English.docx’ contains the ‘Expectations of a Reasonable Patient in Informed Consent in the Singapore Population’ questionnaire that was administered during this study. (DOCX 36 kb)
