# Supplementary material for: Defining reasonable patient standard and preference for shared decision making among patients undergoing anaesthesia in Singapore
Source: BMC Med Ethics. 2017 Feb 2;18:6. doi: 10.1186/s12910-017-0172-2 (PMC5288849; doi:10.1186/s12910-017-0172-2)
Supplement: Additional file 1: — The file titled ‘Questionnaire English.docx’ contains the ‘Expectations of a Reasonable Patient in Informed Consent in the Singapore Population’ questionnaire that was administered during this study. (DOCX 36 kb) [file 12910_2017_172_MOESM1_ESM.docx]

| **Expectations of a Reasonable Patient in Informed Consent in the Singapore Population** | **Date:** |  |
| --- | --- | --- |
|  | **Survey No.:**  **ID/ASA** |  |
| This study aims to understand the expectations of a reasonable patient in the Asian population when taking an informed anaesthetic pre-operative consent. Your participation in this study is greatly appreciated and will benefit future patients in their care.  We appreciate your taking the time to complete the following survey.  The survey should take about 10-15 minutes.  Your responses are voluntary and will be kept confidential. Response will not be identified by individual. All responses will be compiled together and analyzed as a group.  If you have questions about this research study or in the case of any injuries during the course of this study, you may contact the Principal Investigator, Dr. Hairil Rizal at 6326 5519.  If you have questions about your rights as a participant, you can call the SingHealth Centralised Institutional Review Board at 6323 7515 during office hours (8:30 am to 5:30pm).  Thank you.  Dr Hairil Rizal Bin Abdullah  Consultant & Director, Pre-Operative Evaluation Clinic Department of Anaesthesiology  Singapore General Hospital | | |

| **Please check Inclusion and Exclusion criteria prior to starting of survey.** | | | | | |
| --- | --- | --- | --- | --- | --- |
| **Inclusion criteria** | | | **Exclusion Criteria** | | |
| 🞎 Above the age of 21  🞎 Verbal consent for participation taken | | | 🞎 Below age of 21  🞎 Unable to provide informed consent  🞎 Participants with established medical history of dementia | | |
|  | | | | | |
| **PATIENT DEMOGRAPHICS** | | | | | |
| Gender | | 🞎 Male 🞎 Female | | | |
| Age | |  | | | |
| Marital status | | 🞎 Single 🞎 Married 🞎 Divorced 🞎 Widowed | | | |
| Ethnicity | | 🞎 Chinese 🞎 Malay 🞎 Indian 🞎 Caucasian🞎 Others : ________________ (Please specify) | | | |
| Highest Educational Level | | 🞎 None 🞎 Primary School  🞎 Secondary School  🞎 Institute of Technical Education  🞎 Polytechnic  🞎 Junior College  🞎 Undergraduate degree  🞎 Post-graduate degree 🞎 Others: ______________________ (please specify) | | | |
| Household income (monthly) | | 🞎 Retired 🞎 <$3,000 🞎 $3,000-6,000 🞎 $6,000-$9,000  🞎 $9,000-$12,000 🞎 >$12,000 🞎 Unspecified | | | |
| Patient Classification | | 🞎 Private  🞎 Subsidized | | | |
|  | | | | | |
| **SURVEY** | | | | | |
| **1. Below is a list of descriptions of risks and its level of occurrence.  At what level of risk would you consider significant to yourself, and would wish to be informed of?** | | | | | |
| **Level of risk** | **Level of occurrence** | | | **Yes** | **No** |
| 1 in 1 | Something occurring every time | | |  |  |
| 1 in 10 | Risk of something happening to one person in a family | | |  |  |
| 1 in 100 | Risk of something happening to one person in your street | | |  |  |
| 1 in 1000 | Risk of death of any cause at age 40 | | |  |  |
| 1 in 10 000 | Risk of death in a motor vehicle accident in your lifetime | | |  |  |
| 1 in 100 000 | Risk of being murdered | | |  |  |
| 1 in 1 000 000 | Risk of getting electrocuted at home | | |  |  |
| 1 in 10 000 000 | Risk of being struck by lightning | | |  |  |
| **2. The following is a list of possible complications of general anaesthesia and its description for your reference to the next question on the following page.**   \| **Complication** \| **Description** \| \| --- \| --- \| \| Pain on injection of needles \| Prior to surgery, you will experience pain on injection of needles to give medications. \| \| Shivering \| When you wake up in the recovery room, you feel cold and your entire body is shaking uncontrollably. You are unable to hold a cup or speak properly from the shivering. \| \| Nausea \| When you wake up in the recovery room, you feel a desire to vomit, as if you are seasick. Any movement makes it worse. \| \| Sore throat \| When you wake up in the recovery room, your throat is painful, and it hurts to swallow or speak. \| \| Drowsiness and dizziness \| When you wake up in the recovery room, you may feel like the room is spinning or feel sleepy. \| \| Pain at surgical wound site \| When you wake up in the recovery room, you may experience pain that worsens with movement. \| \| Confusion/Delirium \| When you wake up in the recovery room, you may feel confused and agitated; you may feel frightened and see or hear imaginary things \| \| Bruising at intravenous site \| You notice a bruise at the site where injection was attempted; it may be painful on pressing. It usually subsides in days to weeks. \| \| Orodental trauma \| When you wake up in the recovery room, you are told that there was accidental damage to your teeth which will require dentist to see; you may experience a stinging sensation from a cut on your lip. \| \| Heart attack \| You suffer from a heart attack during the operation that may require additional procedures after the operation or intensive care stay \| \| Awareness \| During the surgery, you may be conscious with or without pain but unable to move, and may remember events during the surgery. \| \| Pneumonitis (Lung infection) \| Breathing stomach contents into the lungs can cause lung infections, which may require staying in the Intensive Care Unit. \| \| Nerve damage \| You may wake up experiencing weakness or numbness in certain parts of the body. \| \| Severe drug allergy \| When medications are given, you get a rash with swelling of your face and breathing difficulty. This is usually reversible, but may sometimes be life-threatening. \| \| Stroke/Brain damage \| You wake up with loss of power or sensation. It may be permanent and cause inability to think, feel or move normally. \| \| Blindness/Corneal abrasion \| In the recovery room, you may feel that your vision is blur or may experience pain in your eyes with frequent tearing. \| \| Death \| Your heart stops during the operation and you die in spite of all attempts to revive you \| \| Facial/ Eyelid abrasions \| When you wake up in the recovery room, you feel a stinging sensation over a patch of injured skin on your face (such as cheek, eyelids, chin). \| \| Urinary Retention \| You wake up with difficulty passing urine or abdominal discomfort. You may require insertion of a urinary tube to help you pass urine. \| \| Delayed awakening \| You take much longer than usual to wake up after surgery. May require additional tests and procedures. \| | | | | | |
|  | | | | | |
| **Taking into consideration each complication as described above and its level of risk, kindly answer the following questions: A. Did you already know about this complication prior to today’s consult B. Do you consider this a significant risk and would you wish to be informed?**   \| Level of Risk \| Complication \| A. Did you already know about this complication prior to today’s consult? \| \| B. Do you consider this significant risk and would you wish to be informed? \| \| \| --- \| --- \| --- \| --- \| --- \| --- \| \| **Yes** \| **No** \| **Yes** \| **No** \| \| 1 in 1 \| Pain on intravenous cannulation \|  \|  \|  \|  \| \| 1 in 4 \| Shivering \|  \|  \|  \|  \| \| 2 to 3 in 10 \| Nausea \|  \|  \|  \|  \| \| 1 - 6 in 10 \| Sore throat \|  \|  \|  \|  \| \| 2 to 4 in 10 \| Drowsiness and dizziness \|  \|  \|  \|  \| \| 1 - 3 in 10 \| Surgical wound pain \|  \|  \|  \|  \| \| 1 to 4 in 10 \| Confusion/Delirium \|  \|  \|  \|  \| \| 1 in 10 \| Bruising at intravenous site \|  \|  \|  \|  \| \| 1 in 100 \| Orodental trauma \|  \|  \|  \|  \| \| 4 in 1000 \| Heart attack \|  \|  \|  \|  \| \| 1 in 3000 \| Awareness \|  \|  \|  \|  \| \| 3 in 10,000 \| Pneumonitis (Lung infection) \|  \|  \|  \|  \| \| 1 in 10,000 \| Nerve damage \|  \|  \|  \|  \| \| 1 in 10,000 \| Severe drug allergy \|  \|  \|  \|  \| \| 8 in 100,000 \| Stroke/Brain damage \|  \|  \|  \|  \| \| 1 in 10,000 – 100,000 \| Blindness/Corneal abrasion \|  \|  \|  \|  \| \| 1 in 100,000 \| Death \|  \|  \|  \|  \| \| No exact figures \| Facial/eyelid abrasions \|  \|  \|  \|  \| \| No exact figures \| Urinary Retention \|  \|  \|  \|  \| \| No exact figures \| Delayed awakening \|  \|  \|  \|  \| | | | | | |
| **3. What other complications have you heard and would you like to know about?** ___________________________________________________________________________________________________________________________________________________________________________________________________________________________________________________________________________________________________________________________________________________________________________  **4. To what extent do you wish to be informed of the risks of general anaesthesia?** ☐ No, would rather not know about my risks  ☐ Yes, would want to know about the *dangerous* risks i.e. likely to cause significant injury  ☐ Yes, would want to know the *common* risks i.e. likely to occur  ☐ Yes, would want to know *all possible risks*  **5A. How would you prefer risk disclosure?**  ☐ Verbal description of risk ☐ Actual percentages (e.g. 1 in 1000) ☐ Both  **5B. In what mode would you prefer risk disclosure? You may tick one or more boxes** ☐Pamphlet ☐ Discussion with my anaesthetist ☐ Video ☐ Others: ______________________________  **6. How would you prefer decisions relating to your care be made?** ☐ Would prefer to let the doctor decide after being given basic information on the procedure. The doctor knows what is best for me. ☐ Doctor explains to me about the procedure, benefits and risk and then will let doctor choose and decide. ☐ I would like to make it a joint decision between me and the doctor after a discussion about my illness, procedure, benefits, risk and alternative. ☐ The doctor informs me of the illness, procedure, benefits, risk, alternatives and the doctor's recommendation then I will decide. ☐ I can decide for myself and I will ask the doctor to provide me all information and answer my questions. | | | | | |
